# A Narrative Review of the Evolving Role of Robotic Surgery in Pediatrics: Innovations and Future Prospects

**DOI:** 10.7759/cureus.80419

**Published:** 2025-03-11

**Authors:** Mohammed A E Ibrahim, Mariam Darwish, Reda H Mithany, Andrew Wanees, Mahmoud Elhadidi, Ahmad Elhattab, Nervana M Khalil, Nazeer Ibraheem, Amira Eldesouky, Rezuana Tamanna, Mohamed Ali, Mina H Younan, Michael Shenouda, Amr A Elshahhat, Abdelmoneim Elshamy, Aya E Egeza, Mohamed Elsherbiny, Momen Abdelglil

**Affiliations:** 1 Pediatric Surgery, Manchester University NHS Foundation Trust, Manchester, GBR; 2 Pediatric Surgery, Mansoura University Children's Hospital, Mansoura, EGY; 3 Colorectal Surgery, Torbay and South Devon NHS Foundation Trust, Torquay, GBR; 4 General Surgery, Royal Devon University Healthcare NHS Foundation Trust, Exeter, GBR; 5 Urology, The Royal Wolverhampton NHS Trust, Wolverhampton, GBR; 6 General Surgery, Watford General Hospital, Watford, GBR; 7 General Surgery, Bronglais Hospital, Aberystwyth, GBR; 8 General Surgery, Hull University Teaching Hospitals NHS Trust, Hull, GBR; 9 General Surgery, Kasr Alainy Hospital, Cairo, EGY; 10 Orthopedics, Mansoura University Children's Hospital, Mansoura, EGY; 11 General Surgery, Zagazig University, Zagazig, EGY; 12 General Surgery, Mansoura University Children's Hospital, Mansoura, EGY

**Keywords:** ai, pediatric surgery, robotic in surgery, surgical innovation, surgical robotics

## Abstract

Robot surgery has significantly improved surgical interventions for pediatric patients by enhancing surgical precision, minimizing complications, and improving overall patient outcomes. Over the past few years, substantial advancements in technology and surgical techniques have facilitated the widespread adoption of robotic systems in pediatric surgical procedures across multiple specialties. These encompass specialties such as pediatric urology, general surgery, thoracic surgery, and oncology, contributing to its adoption and widespread implementation in clinical practice. The integration of robotic platforms has enabled surgeons to perform complex procedures with greater dexterity, improved visualization, and enhanced control. This comprehensive review aims to provide an in-depth analysis of the evolution of robotic surgery, its current applications in pediatric surgery, its advantages over conventional surgical techniques, and the potential limitations and challenges associated with its usage and generalization in clinical practice.

## Introduction and background

The robotic surgical intervention provides several benefits compared to traditional methods, including greater precision, reduced hazards, and superior visualization in tight surgical spaces. These benefits are especially important in pediatric patients with more pronounced anatomical constraints [1]. The da Vinci Surgical System and newer platforms like the Senhance Surgical System are now used for various pediatric operations. These systems offer enhanced visualization, dexterity, and accuracy, which are crucial when operating on smaller anatomical structures [2]. Despite the advancement of robotic systems, challenges remain in pediatric robotic surgery. The high cost of robotic systems and instruments specifically designed for children continues to be a barrier to global use. Additionally, there is still a lack of extensive clinical data supporting the long-term outcomes of robotic surgeries in pediatric patients [3]. The use of robotic surgery in neonates presents distinct challenges, including limited operative space, difficulty in trocar placement, and an increased risk of instrument collision [4]. With technological advancements and increasing surgical expertise, robotic surgery will likely become more viable for pediatric patients [5].

## Review

Methodology

This review examined published research articles on robotic surgery in pediatric patients, focusing on studies published between 2016 and 2025. A comprehensive search was conducted across multiple databases using keywords including ("robotic pediatric surgery" OR "robotic surgery in children") AND ("clinical outcomes" OR "cost-effectiveness" OR "learning curve" OR "artificial intelligence").

We included articles if they were peer-reviewed, published within the last 10 years, and focused on robotic surgery in pediatric patients. Articles unrelated to pediatric surgery or lacking original clinical data were excluded. Articles were critically assessed for relevance to the study objectives. Key data on surgical benefits, emerging robotic technologies, ethical considerations, and future directions were extracted and analyzed to provide an updated perspective on the role of robotics in pediatric surgery.

Overview of robotic surgery in pediatrics

Robotic surgery has transformed surgical practices for patients. Robotic surgery for adults began in the mid-1980s, using systems such as the PUMA 560 and the da Vinci Surgical System, which gained FDA approval in 2000 [6].

Since its inception, pediatric robotic surgery has achieved several significant milestones, including advancements in instrument miniaturization, improved surgical precision, expanded procedural applications, and enhanced patient outcomes. The first documented pediatric robotic procedure was conducted in 2001 when a girl had a robotic Nissen fundoplication. Over the years, various procedures have been successfully performed using robotic assistance, including pyeloplasty, fundoplication, and other complex procedures previously challenging due to limited working space and instrument size constraints in children [6].

Recent studies indicate that robotic surgeries in children have comparable outcomes to traditional laparoscopic methods while offering benefits such as shorter hospital stays and reduced complications [3].

Robotic diaphragmatic hernia repair

Robotic transthoracic diaphragmatic hernia repair has emerged as an innovative and effective approach with several advantages, including enhanced visualization, improved dexterity, and minimally invasive access to the thoracic cavity [7,8].

Holder and Bakeer published a case report demonstrating the successful use of robotic surgery for a delayed traumatic diaphragmatic hernia. The authors utilized the da Vinci Surgical System to reduce a substantial part of the stomach body that herniated into the left hemithorax. The diaphragmatic defect was repaired using tension-free primary closure without mesh reinforcement, followed by gastropexy. This case demonstrated the advantages of robotic surgery in addressing intricate anatomical challenges [9].

Another study on Morgagni hernia repair described a robotic transabdominal preperitoneal technique. The robotic platform's wristed instruments facilitate navigation in small spaces, offering advantages over laparoscopy [10].

While robotic diaphragmatic hernia repair offers significant advancements, it is important to report that its global usage may be limited by factors such as access to robotic platforms, high costs, and the need for specialized expertise [11].

Robotic repair of congenital esophageal atresia

Esophageal atresia is a birth defect where the esophagus is discontinuous, preventing the connection between its parts [12]. Robotic thoracoscopic surgery is a minimally invasive approach that offers enhanced dexterity, accuracy, and 3D visualization depending on the da Vinci Surgical System. It enables good and accurate movements and better control during thoracoscopic surgery [13].

Several reported cases of successful robotic repair of esophageal atresia have been published. Li et al. reported the first case of robotic thoracoscopic esophageal anastomosis in China, demonstrating the feasibility of this approach. The patients' mean weight was 3.2 kg, and the operative times were 95 minutes, with the anastomotic time being 27.49 minutes. Follow-up duration was 12 months, and tracheoesophageal fistula recurrence was documented in one case [4].

However, the risk of esophageal fistula recurrence remains a challenge, and there is a need for specialized training for surgeons. As technology advances, further miniaturization of robotic instruments and improvements in training programs will likely enhance the feasibility and success of robotic repair of EA in neonates [14].

Pediatric and neonatal colorectal surgeries

Robotic surgery is gaining recognition as a safe approach for treating pediatric Hirschsprung's disease. This procedure typically involves using three robotic arms and an additional 5-mm trocar. Studies report promising outcomes, including an average operative time of 93.2 ± 35 minutes and minimal blood loss. Postoperative results indicate that 74.6% of patients experienced one to two bowel movements every day, along with low rates of complications such as fecal incontinence (5.4%), enterocolitis (7.3%), and mild soiling (3.6%) [15].

The learning curve for robotic surgery in Hirschsprung's disease seems shorter than that for open or laparoscopic techniques, though achieving optimal results still requires a skilled surgical team and tailored procedural adjustments [1].

Compared to conventional laparoscopic surgery (CLS) and transumbilical laparoendoscopic single-site surgery (TU-LESS), robotic surgery for Hirschsprung's disease has distinct differences. Although robotic surgery requires a longer operation time than CLS and TU-LESS, TU-LESS provides the best aesthetic outcomes, followed by CLS and robotic surgery. However, no significant differences have been observed among the three techniques in terms of recovery time of digestive function or postoperative complications [1].

Lung lobectomy for congenital lung lesions

Congenital pulmonary airway malformation and intralobar pulmonary sequestration are pediatric thoracic surgery's most common lung diseases [16]. Lobectomy is the standard surgical approach for these conditions [17].

Performing minimally invasive pulmonary resection in children poses significant challenges, regardless of whether it is done with robotic assistance or a thoracoscopy. Robotic lobectomy has been gaining wider acceptance since Ashton et al. and Morgan et al. independently performed their pioneering procedures in 2003 in adult patients in the United States [18,19].

Li et al. conducted a retrospective study comparing robotic and thoracoscopic pulmonary resection using the da Vinci Surgical System; 29 robotic pulmonary resection and 42 thoracoscopic pulmonary resection cases were performed, with three requiring conversion to thoracotomy. The study found robotic surgery a safe and feasible option for patients over six months old, improving surgeon efficiency and comfort through advanced ergonomics and 3D visualization [20].

Use of robotics in urological disorders

Robotic Ureteral Reimplantation in Children With Vesicoureteral Reflux

Vesicoureteral reflux (VUR) increases the incidence of urinary tract infections and renal scarring. Diagnosis is challenging, especially in infants, due to the non-specific symptoms [21-23].

The main goal of VUR management is to reduce renal scarring by reducing infection. In addition, clinicians aim to prevent UTIs. Management may be non-surgical, minimally invasive (endoscopic injection), or surgical (ureteral reimplantation) [24-26].

Robot-assisted ureteric reimplantation (RALUR) for VUR offers several benefits, including enhanced visualization through 3D imaging, reduced postoperative pain, and shorter hospital stays, typically lasting one to two days. This minimally invasive approach improves cosmetic outcomes with smaller incisions and comparable success rates to traditional open surgery, achieving up to 94% reflux resolution. Additionally, RALUR minimizes complications such as hematuria and bladder spasms, making it a favorable option for complex VUR cases [26-28].

Ureteropelvic Junction Obstruction

Robot-assisted pyeloplasty (RALP) has emerged as the gold standard for treating pediatric ureteropelvic junction obstruction (UPJO), offering shorter operative times and success rates comparable to laparoscopy. A systematic review (2012-2022) highlighted its advantages, except in small infants, where open surgery is preferable. Since 2009, RALP has become the preferred approach, proving safe and effective, even in redo procedures and complex cases, with growing adoption worldwide [29].

Pediatric RALP has a success rate of 90-100%, offering shorter hospital stays and reduced postoperative pain compared to open or laparoscopic approaches. Operative times are improving, especially in high-volume centers. Early complications include fever, pain, hematuria, urinary leaks, and stent migration, while late complications, though rare, include ureteral stenosis, urinary leakage, and stone formation. RALP is a safe and effective treatment for UPJO [30].

The robotic retroperitoneoscopic approach for UPJO has emerged as a technique in urologic surgery, combining the accuracy and precision of robotic assistance with the anatomic benefits of retroperitoneal access. Recent studies demonstrate its superiority over laparoscopy, including reduced anastomotic times, fewer complications, and enhanced suturing accuracy, particularly in pediatric and complex anatomical cases [31-34].

Robotics in Bladder Reconstruction

**Figure 1:**
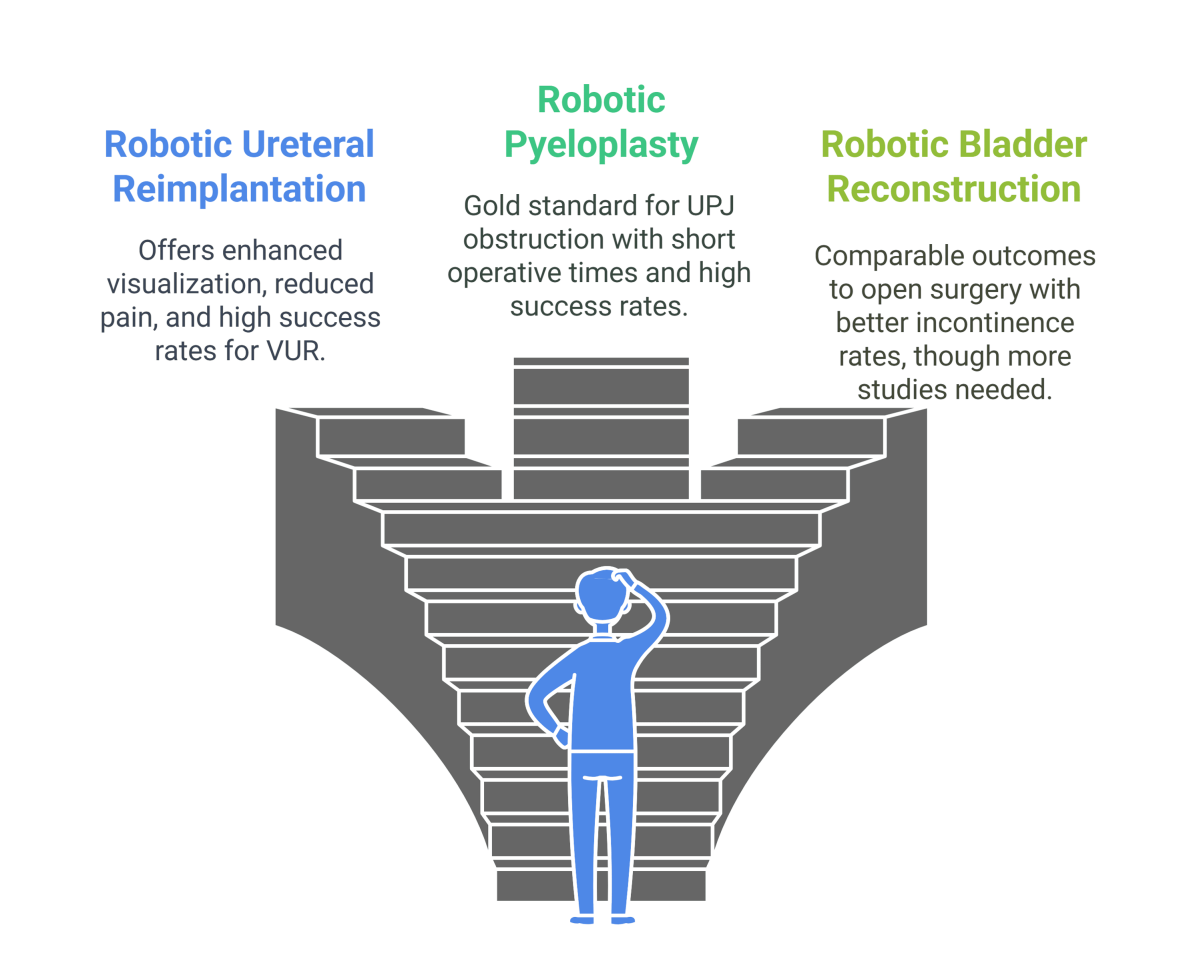
Various applications of robotic surgery in urological disorders VUR: vesicoureteral reflux, UPJ: ureteropelvic junction Image Credit: Momen Abdelglil Source: [21-35]

Over the past five years, robotic bladder neck reconstruction has been increasingly used and documented in the literature, primarily through case series. Existing evidence, encompassing various reconstructive techniques for patients with different underlying diseases and conditions, indicates that the outcomes are comparable to those of open surgery. At the same time, incontinence rates appear to be lower. However, further prospective studies with extended follow-up periods must validate these results and assess their long-term durability (Figure 1) [35].

Role of robotic surgery in oncological conditions

Robotic surgery is gaining acceptance as a good alternative to the open type for pediatric tumor removal, particularly in neuroblastoma and Wilms' tumor. While open surgery remains the gold standard, robotic techniques' potential benefits drive their adoption [36].

A successful robotic resection of a stage IV neuroblastoma has been documented, with the authors attributing the positive outcome to the enhanced visualization and precision the robotic system provides. They suggested that these capabilities facilitated a more thorough dissection and skeletalization of the tumor's vasculature than would likely have been achievable with a laparoscopic approach [37]. In another instance, a 15-year-old girl with a 4 cm juvenile cystic adenomyoma underwent successful robotic resection. The authors highlighted the improved ergonomics of the robotic platform, which facilitated a meticulous four-layered closure of the uterus. The patient subsequently experienced an uncomplicated postoperative recovery [38].

Some concerns have been raised about the use of robotic surgery in children, specifically regarding adherence to fundamental oncological principles. These concerns center on achieving clear resection margins and preventing tumor spillage. One theory suggests that the lack of haptic feedback in robotic procedures may hinder the surgeon's ability to distinguish between cancerous and healthy tissue [39]. However, some argue that the enhanced visualization provided by the robotic system may offset this limitation [40].

A critical analysis emphasized the importance of appropriate patient selection for robotic surgery in pediatric oncology to ensure favorable oncological outcomes while minimizing surgical morbidity [5].

**Figure 2:**
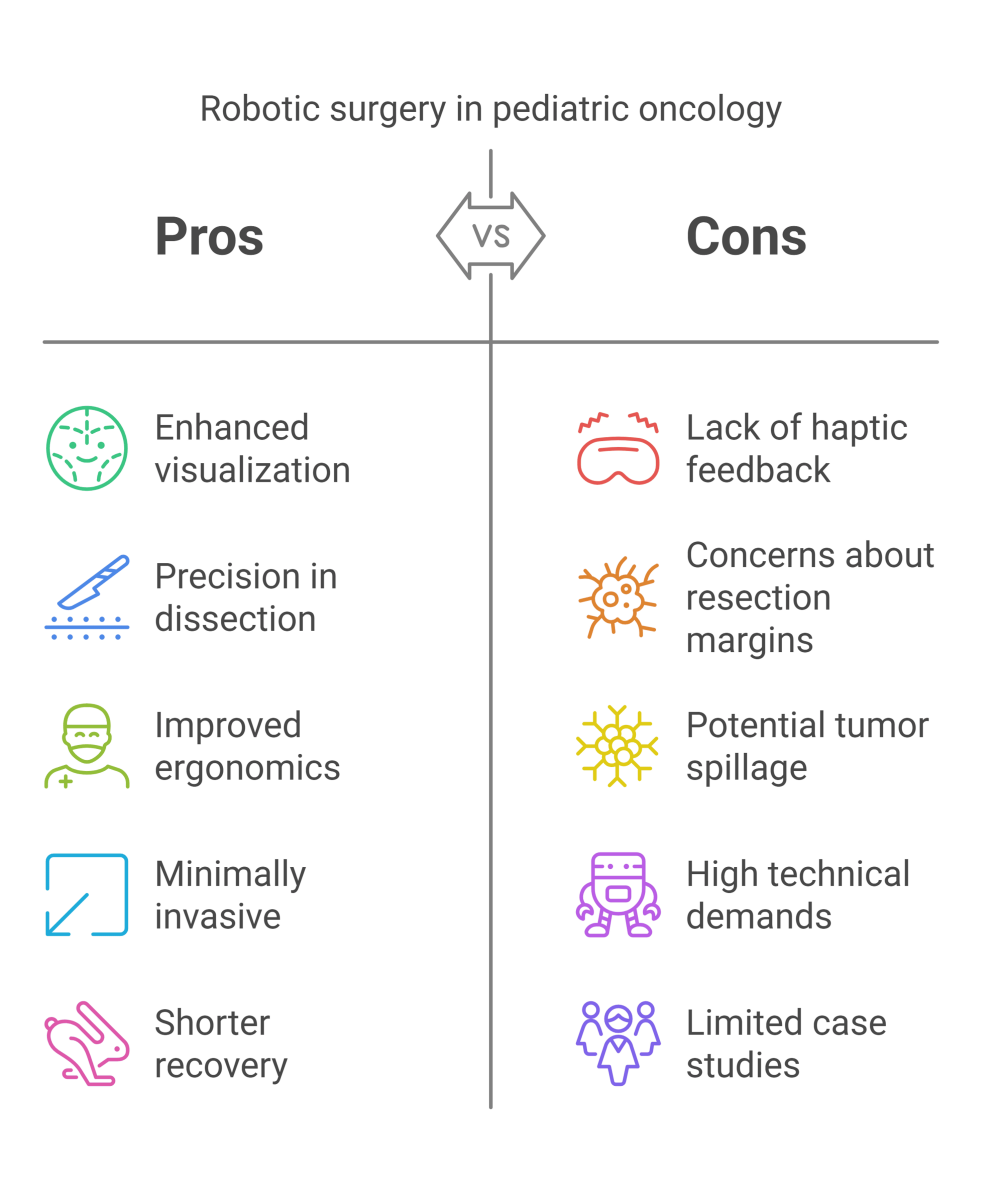
Robotic surgery in pediatric oncological conditions Image Credit: Momen Abdelglil Source: [36-40]

Robotic adrenalectomy is emerging as a minimally invasive alternative for pediatric adrenal tumors, with a high success rate of approximately 91.9%. While comparable to open surgery, it presents specific challenges, with few cases requiring conversion due to complications or technical difficulties [2]. Studies have shown that patients undergoing robotic procedures, such as adrenalectomy, often experience shorter hospital stays and a faster return to normal activities compared to those undergoing traditional surgical methods (Figure 2) [2].

Training and learning curve of robotic surgery in pediatric

Surgeons should receive simulation training before conducting robotic pediatric surgeries to gain skills. This may be possible using virtual reality (VR), dry labs with models, wet labs with cadavers, or live anesthetized animals for safe procedure practice [41].

In 2006, SAGES-MIRA developed guidelines for robotic surgery training and credentialing, recommending a combination of didactic courses, hands-on training, and supervised operating room experience to enhance learning [42]. Fundamentals of Robotic Surgery and da Vinci Surgical System training provide didactic-only foundational education in robotic surgery, used alone or with VR systems. More comprehensive programs, including the SAGES Robotics Masters Series, Robotics Training Network, and Fundamental Skills of Robotic-Assisted Surgery, integrate didactic sessions with hands-on robotic simulation and cadaver courses. VR systems function as standalone simulators but can complement didactic training [43].

Structured training programs that combine VR simulations, practical experience, and mentorship are crucial to reducing the steep learning curve associated with robotic systems [44]. VR platforms help trainees become more proficient in a safe setting by simulating actual surgical situations without the dangers of real patients. Furthermore, receiving mentoring from senior surgeons offers insightful criticism and direction, which aids newcomers in improving their methods [45].

Challenges and limitations of robotic surgeries

One of the most pressing issues in robotic surgery is the mismatch between the size of robotic instruments and the small working space of children. The da Vinci Surgical System, a commonly used robotic platform, recommends a 6 cm distance between ports, which is often unachievable in pediatric patients due to their smaller body size [46,47].

This spatial constraint severely limits the maneuverability of robotic arms within the confined operative field. Furthermore, the current robotic instruments, typically 5 mm or 8 mm in diameter, are often too large [48].

Traditional laparoscopic instruments used in pediatric surgeries can be as small as 3 mm, highlighting the need for smaller robotic tools specifically designed for neonatal use. The lack of commercially available 3 mm robotic instruments significantly limits the application of robotic surgery in children and very young infants [49].

Robotic surgery faces challenges in neonates, especially those under 3.0 kg, due to the large size of instruments and limited working space. These constraints can cause robotic arm collisions, reducing precision and increasing the risk of complications, sometimes requiring conversion to open surgery [47].

Technical Challenges and Instrument Design

The learning curve of robotics is exacerbated by the unique challenges posed by neonatal anatomy and the need for specialized techniques to navigate the limited working space [49].

Cost and Accessibility

The high cost of these systems is a significant problem, and it can be difficult for pediatric hospitals to afford their application. Additionally, robotic surgery tends to increase the overall cost of procedures compared to traditional methods like laparoscopic or open surgeries [1].

Specialized training for doctors and staff adds to costs. Additionally, the smaller market for pediatric devices discourages investment in neonatal-specific robotic instruments, hindering progress and accessibility [50].

Accountability and Legal Liability

As robotic systems become more involved in surgeries, determining accountability for adverse outcomes becomes more complex, involving the surgeon, hospital, and manufacturer. There is no clear consensus on the legal standing of robotic surgery in children, which can lead to disputes in litigation. The misconception that robotic surgery absolves the surgeon of legal responsibility must be corrected. Clearer guidelines and laws are needed to address the unique medicolegal issues, including mechanical failures, malfunctions, and the potential overreliance on machines that may overshadow the surgeon’s role [51].

Ethical Considerations and Informed Consent

**Figure 3:**
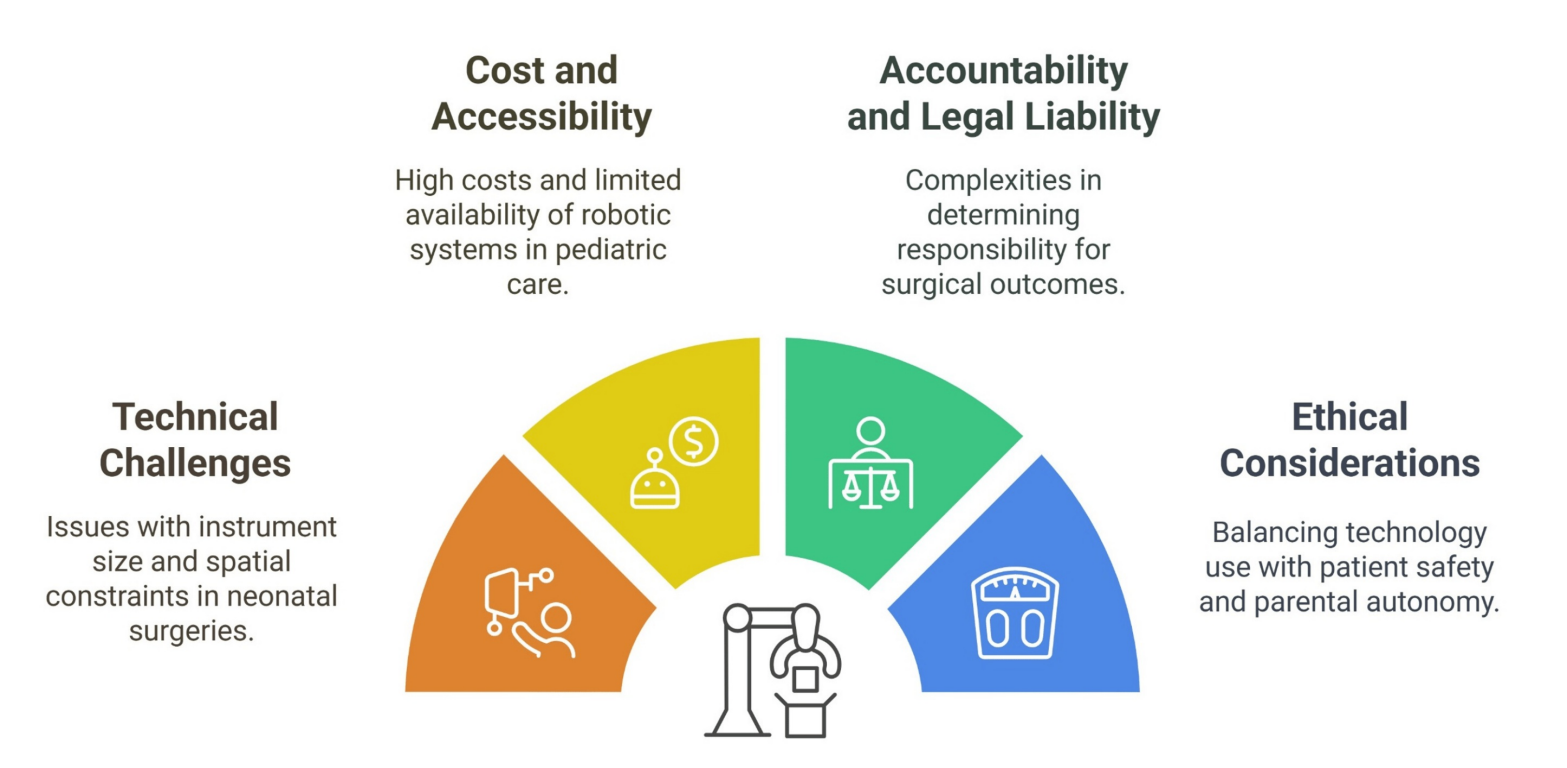
Complexities and challenges of the global application of robotic surgery Image Credit: Momen Abdelglil Source: [46-52]

The ethical implications of using advanced technology on vulnerable patients are significant in pediatric and neonatal robotic surgery. Informed consent becomes complex as decision-making lies with guardians. Surgeons must facilitate shared decision-making, balancing patient safety and respecting parental autonomy. The informed consent process should include the surgeon’s experience, known outcomes, and the innovative aspects of the procedure. Ongoing oversight is necessary, per the American Academy of Pediatrics’ guidelines. The ethical framework should also prioritize beneficence, nonmaleficence, and justice, ensuring that the benefits of the technology outweigh the risks and that access is equitable (Figure 3) [52].

Future perspectives

Robotic surgery initially gained traction with prostatectomy in adults, later expanding to pediatric procedures such as pyeloplasty. The advantages of robotic surgery include 3D visualization, tremor elimination, a fourth arm, and a 7-degree range of motion, particularly benefiting anastomotic suturing. Its use in pediatric urology includes ureteral reimplantation and complex reconstructive procedures. However, the da Vinci Surgical System lacks haptic feedback, complicating the learning process. New robotic systems, like the Senhance Surgical Robotic System, offer better maneuverability, individual robotic arm carts, magnet-based instrument attachment for quicker intraoperative exchanges, and compatibility with smaller ports [53].

## Conclusions

Since its introduction in the United States, robotic surgery has transformed surgical practice by enhancing precision, reducing complications, and shortening hospital stays. Pediatric surgery offers improved dexterity, superior visualization, and greater control, leading to better clinical outcomes. However, challenges such as high costs, limited accessibility, and the need for specialized training have hindered widespread adoption. With ongoing technological advancements, refined techniques, and expanded training programs, robotic surgery is expected to become more feasible and widely implemented, offering a safer and more effective approach for complex pediatric procedures.
